# Access to Cleaning Services Alters Fish Physiology Under Parasite Infection and Ocean Acidification

**DOI:** 10.3389/fphys.2022.859556

**Published:** 2022-06-08

**Authors:** José Ricardo Paula, Tiago Repolho, Alexandra S. Grutter, Rui Rosa

**Affiliations:** ^1^ Swire Institute of Marine Science, School of Biological Sciences, The University of Hong Kong, Hong Kong, Hong Kong SAR, China; ^2^ MARE—Marine and Environmental Sciences Centre, Laboratório Marítimo da Guia, Faculdade de Ciências, Universidade de Lisboa, Cascais, Portugal; ^3^ School of Biological Sciences, The University of Queensland, St. Lucia, QLD, Australia

**Keywords:** cooperation, cleaning mutualism, ocean acidification, climate change, metabolism, adaptation

## Abstract

Cleaning symbioses are key mutualistic interactions where cleaners remove ectoparasites and tissues from client fishes. Such interactions elicit beneficial effects on clients’ ecophysiology, with cascading effects on fish diversity and abundance. Ocean acidification (OA), resulting from increasing CO_2_ concentrations, can affect the behavior of cleaner fishes making them less motivated to inspect their clients. This is especially important as gnathiid fish ectoparasites are tolerant to ocean acidification. Here, we investigated how access to cleaning services, performed by the cleaner wrasse *Labroides dimidiatus*, affect individual client’s (damselfish, *Pomacentrus amboinensis*) aerobic metabolism in response to both experimental parasite infection and OA. Access to cleaning services was modulated using a long-term removal experiment where cleaner wrasses were consistently removed from patch reefs around Lizard Island (Australia) for 17 years or left undisturbed. Only damselfish with access to cleaning stations had a negative metabolic response to parasite infection (maximum metabolic rate—*Ṁ*O_2Max_; and both factorial and absolute aerobic scope). Moreover, after an acclimation period of 10 days to high CO_2_ (∼1,000 µatm CO_2_), the fish showed a decrease in factorial aerobic scope, being the lowest in fish without the access to cleaners. We propose that stronger positive selection for parasite tolerance might be present in reef fishes without the access to cleaners, but this might come at a cost, as readiness to deal with parasites can impact their response to other stressors, such as OA.

## Introduction

Parasite infection can adversely impact fish. When a parasite feeds on host tissues and resources, it alters the host physiological states, compromising activities that require energy expenditure (Sheldon and Verhulst, 1996; Lochmiller and Deerenberg, 2000). Specifically, ectoparasites can impact the hosts’ energy budgets by affecting the metabolism (inducing higher resting metabolic rate), growth, and reproduction (Fenton and Rands, 2006; Jones and Grutter, 2008; Grutter et al., 2011). On coral reefs, dedicated cleaner fishes provide cleaning services by eating ectoparasites off the bodies of other “client” fishes (Grutter, 1999; Vaughan et al., 2017). The cleaner wrasse, *Labroides dimidiatus,* is the most ubiquitous species of cleaner in the Indo-Pacific region. This cleaner engages in more than 2,200 interactions with clients, eating more than 1,100 ectoparasites a day (Grutter, 1996). Analysis of cleaners’ stomach contents indicates that these cleaners mostly eat body-fluid feeding gnathiid isopod ectoparasites, removed during their cleaning interactions with clients (Grutter, 1996; Grutter, 1997). In addition to their effect on host metabolism, gnathiids can lower the blood volume of their hosts (up to 85% from young damselfish, *Pomacentrus amboinensis*) (Grutter et al., 2011), transmit blood-borne parasites (Hayes et al., 2011a), and in large numbers, can even kill the adult fish (Hayes et al., 2011b). Since cleaners can consume most of the daily emerged gnathiids on a reef per day, access to cleaning services represents a substantial advantage to clients, as cleaners can directly remove the parasites from client surfaces and control gnathiid populations by reducing the parasite infection rates (Grutter, 2008; Grutter et al., 2018). Therefore, it is no surprise that the presence of *L. dimidiatus* is associated with the increased client condition, growth and body size, and influences fish abundance, biodiversity, and juvenile recruitment (Bshary, 2003; Grutter et al., 2003; Clague et al., 2011; Ros et al., 2011; Waldie et al., 2011; Sun et al., 2015; Wagner et al., 2015).

Reef fishes can be vulnerable to ocean acidification (OA) caused by a decrease in seawater pH by the absorption of anthropogenic CO_2_ emissions. High CO_2_, and thus the associated lower pH, has been documented to impact behavior traits such as activity, homing, anxiety, learning, lateralization, and olfactory and auditory systems (reviewed by Clements and Hunt, 2015). Recent studies have also documented low or no effect of OA on fish behavior (Sundin et al., 2017; Raby et al., 2018; Clark et al., 2020), suggesting the species-specific variability in fish behavioral responses to OA. In addition, cleaning mutualisms are not immune to OA. After prolonged exposure to OA, interactions between the cleaners and clients are reduced and the cleaners lose their motivation to engage in cleaning interactions along with a disruption in interaction quality through possible neurobiological disruption (Paula et al., 2019a) and interaction-derived cognitive performance (Paula et al., 2019b). Within this context, the client fishes’ ability to reduce ectoparasite loads diminishes due to lower cleaner motivation, along with the resulting potential increase in the infection rates onto fish (Grutter et al., 2018), as reduced cleaning activity also may not control local gnathiid population densities (Sikkel et al., 2019). However, it is also possible that after the long-term lack of access to cleaning services, surviving client fishes might be physiologically adapted to deal with ectoparasite infections. Moreover, a previous study showed that gnathiid survival is not affected by laboratory acclimation to high CO_2_, suggesting that gnathiids can tolerate ocean acidification (Paula et al., 2020).

High CO_2_ has also been documented to impact the reef fish physiological traits such as escape performance (Allan et al., 2013; Allan et al., 2014), metabolism (Wisenden, 2012; Couturier et al., 2013; Rummer et al., 2013; McMahon et al., 2020), and reproduction (Miller et al., 2013; Welch and Munday, 2016). A meta-analysis of fish metabolic responses to OA revealed that there is an increase on resting metabolic rate (*Ṁ*O_2Rest_), but there seems to be no overall effect of OA on fish absolute aerobic scope (*Ṁ*O_2Max_ - *Ṁ*O_2Rest_) (Hannan and Rummer, 2018). Metabolic rate can be measured during resting (resting metabolic rate—*Ṁ*O_2Rest_), giving us information on the basal metabolic costs of an organism to perform life-sustaining functions. During maximum exercise, metabolic rate measures the maximum rate of aerobic metabolism (maximum metabolic rate—*Ṁ*O_2Max_) an animal can reach, and it is related to the maximum rate of oxygen transport, extraction, and use (Hannan and Rummer, 2018; Hasley et al., 2018). Aerobic scope represents the animal’s capacity to increase its aerobic metabolic rate above the maintenance levels (i.e., the difference between *Ṁ*O_2Max_ and *Ṁ*O_2Rest_), being used as proxy for physiological fitness (Clark et al., 2013). Aerobic scope can be presented in absolute terms (i.e., *Ṁ*O_2Max_ - *Ṁ*O_2Rest_) or as factorial aerobic scope (i.e., *Ṁ*O_2Max_/*Ṁ*O_2Rest_). These measures indicate an absolute or proportional increase, respectively, in the oxygen consumption rates that an animal can reach above the baseline levels (Clark et al., 2013). Measuring and presenting these different metabolic variables in the context of environmental stress research is crucial to understand how a given stressor (such as OA) can add a cost to the basal life-sustaining functions (*Ṁ*O_2Rest_), impact oxygen transport (*Ṁ*O_2Max_), and affect physiological fitness. So far, only four studies have reported a decreased in aerobic scope (along with lower *Ṁ*O_2Max_; Methling et al., 2013; Tirsgaard et al., 2015; or no differences in *Ṁ*O_2Max_ and *Ṁ*O_2Rest_; Hamilton et al., 2017) and of those only one used CO_2_ levels predicted to occur at the end of the century (along with higher *Ṁ*O_2Rest_; Munday et al., 2009). In contrast, 19 studies have reported no effects on aerobic measures of performance and in three studies performance increased over 12 different fish species (reviewed by Hannan and Rummer, 2018). One explanation for these conflicting results is that there is a species-specific response to high CO_2_.

Since the cleaners’ motivation to interact with clients drops with OA (Paula et al., 2019a), one can conceive a future scenario where client fishes must deal with the physiological impacts of OA together with the stress induced by ectoparasitism, without having access to a way to remove parasites (i.e., cleaning interactions). To understand how access to cleaning interactions can modulate fish physiological responses to parasitic infection and OA, we used the small resident ambon damselfish (*Pomacentrus amboinensis*) collected from a long-term cleaner removal experiment on Lizard Island (Australia) maintained since 2000 (Waldie et al., 2011; Grutter et al., 2019). In this long-term experiment, 18 isolated patch reefs have been manipulated so that half had all juvenile and adult *L. dimidiatus* removed around every 3 months over 17 years, whereas the other reefs were left unmanipulated as controls. This created habitats where fish either have no access to cleaning interactions and a potentially higher exposure to ectoparasites, or access to cleaning with a potentially lower parasite exposure (Grutter et al., 2018). Since this long-term experiment has been running longer than the lifespan of this damselfish [approximately 6.5 years (McCormick, 2016)], individuals from “cleaner-absent” reefs had no access to cleaners and possibly had higher ectoparasite exposure for their lifetime. Importantly, since each reef is separated by at least 5 m of sand, these territorial resident damselfish do not cross between the reefs once juveniles have settled on the reef (Binning et al., 2018). We combined this long-term experiment with a 10-days laboratory exposure to high CO_2_ and examined whether living on reefs with or without the access to cleaner wrasses (*L. dimidiatus*) influences clients’ (*P. amboinensis*) metabolic responses under OA (the predicted conditions of ocean acidification for 2100, ∼1,000 μatm, RCP 8.5 scenario; Bindoff et al., 2019). Moreover, we also performed an experimental parasite infection assay using the cultured gnathiid isopods. This allowed us to test the effects of CO_2_ treatment, cleaner presence, and parasite infection on client fish aerobic metabolism, namely resting and maximum metabolic rate, and aerobic scope (both factorial and absolute aerobic scope are presented and analyzed, providing transparency as suggested in Halsey et al., 2018).

## Material and Methods

### Cleaner Fish Removal Experiment

Our study was conducted using fish from eight small spatially isolated patch reefs in shallow (3–7 m deep) areas in Lagoon and Casuarina Beach of Lizard Island (14°40S, 145°28′E), Great Barrier Reef, Australia, during the austral winter (August-September 2017). These reefs are part of a larger ongoing long-term cleaner fish removal experiment initially involving 18 reefs in total (12 at Lagoon and 6 at Casuarina Beach, then reduced to 16 reefs, see Waldie et al., 2011; Sikkel et al., 2019). At the time of the study, the reefs were allocated randomly into seven removal (“cleaner-absent,” *n* = 7, average area ± SE: 131 ± 34 m^2^) and nine control reefs (“cleaner-present,” *n* = 9, average area ± SE: 134 ± 17 m^2^). Since September 2000, after the removal of the initial cleaners, these reefs were inspected at approximately 3-month intervals for the presence of cleaner wrasses. In the cleaner-absent reefs, any new cleaner recruit was removed by hand- and barrier nets, whereas on the cleaner-present reefs they were counted (see Supplementary Figure S1 for numbers of cleaners encountered from 2000 to 2017). Following the 2016/2017 mass bleaching event at Lizard Island, along with the northern portion of the Great Barrier Reef (Hughes et al., 2017), four of the nine control cleaner-present reefs lost their cleaners (Sikkel et al., 2019). Here, we only used fish from the four cleaner-present reefs that had not lost their cleaners during the bleaching event.

### Fish Capture and CO_2_ Exposure

**FIGURE 1:**
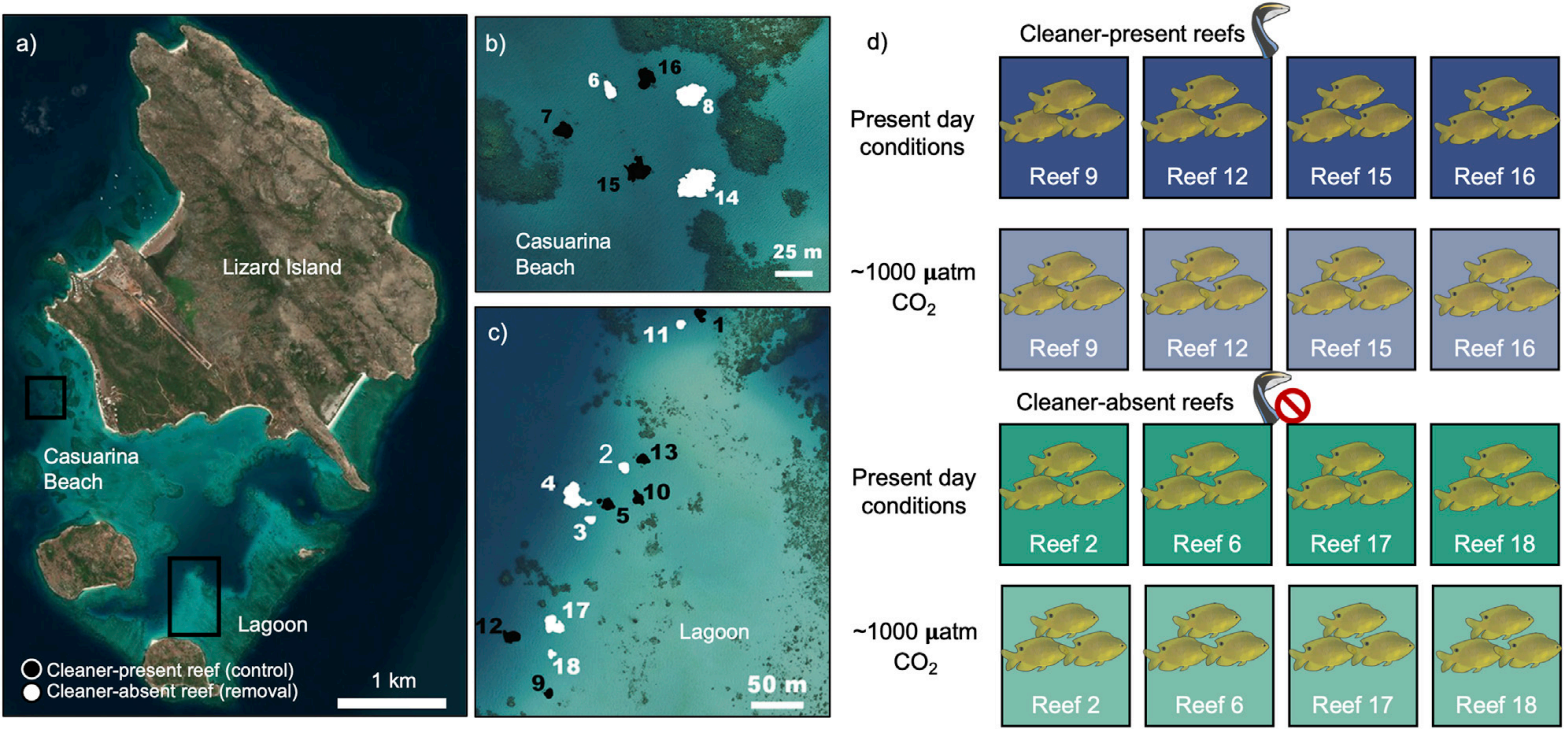
Experimental design used in this study. On the maps **(A–C)**, white represent reefs where cleaner fish *Labroides dimidiatus* were removed throughout 17 years, black represent control reefs. **(D)** *Pomacentrus amboinensis* were collected from reefs 9, 12, 15, and 16 (control reefs) and reefs 2, 6, 17, and 18 (removal reefs) and acclimated to one of two conditions for 20 days: Control (∼350 µatm pCO_2_) or ocean acidification (High CO_2_, ∼1,000 µatm pCO_2_). Map adapted from Grutter et al. (2019).

To investigate whether the presence of cleaner wrasses affected the clients’ physiological responses to ocean acidification and parasite infection, we collected 96 individuals (*P. amboinensis*; 12 per reef; SL = 4.63 ± 0.12 cm, weight = 4.78 ± 0.34 g, and mean ± SE), 48 from the cleaner-present reefs (reefs 9, 12, 15, and 16, Figure 1) and other 48 from four randomly selected cleaner-absent reefs (reefs 2, 6, 17, and 18, Figure 1). Among the initial 96 fish captured, three died during the acclimation due to unknown reasons (one in high CO_2_/cleaner-present; one in high CO_2_/cleaner-absent; and one in control CO_2_/cleaner-present). After 4 days of acclimation to the experimental tanks at Lizard Island Research Station, we exposed them for 10 days to one of the two treatments, control water (present-day CO_2_ ∼ 350 µatm CO_2_), or the predicted conditions of ocean acidification for 2100, following the Representative Concentration Pathway (RCP) 8.5 scenario from the Intergovernmental Panel on Climate Change (IPCC; high CO_2_ ∼ 1,000 µatm CO_2_; Bindoff et al., 2019) in a crossed factorial design (*n* = 24—control/cleaner-absent; *n* = 23—control/cleaner-present *n* = 23—high CO_2_/cleaner-absent; *n* = 23—high CO_2_/cleaner-present; see Figure 1). Initial exposure day among fish varied randomly over 12 days, as the aerobic metabolism analysis (done at day 11, after 10 complete days of exposure) took 12 days to complete. This ensured that all fish had the same exposure duration to CO_2_. This totaled 23 days from the exposure of the first batch of four fish to the metabolism analysis of the last batch of four fish. The exposure time is in line with several other ocean acidification studies performed with the same species (Munday et al., 2009; Heuer et al., 2016; Munday et al., 2016). The fish were maintained in one of the eight flow-through systems (4 fish per CO_2_ treatment) where natural seawater was pumped from the sea into three 10,000 L seawater storage tanks. From these, the seawater was supplied to a mixing tank (flux: 270 L h ^−1^; volume: 130 L) and from these to each experimental tank (flux 12 L h ^−1^; volume: 10 L). The fish were provided with a polyvinyl chloride (PVC) tube for refuge (4 cm diameter, 10 cm length). pCO_2_ control was performed indirectly by adjusting the pH to a nominal value defined by CO2SYS software using measured salinity, total alkalinity, temperature, and desired pCO_2_ as input variables. Levels of pH_NBS_ were automatically adjusted (Profilux 3.1N, GHL, Germany), downregulated by direct injection of CO_2_ gas (BOC, Australia), and upregulated through aeration with atmospheric air in mixing tanks. Seawater temperature was maintained similar to the current reef temperature due to the flow of recently captured seawater. We used handheld equipment to complement the automatic systems to monitor and record seawater parameters, measuring seawater temperature (Hanna CheckTemp 1C, Portugal), salinity (V2 refractometer, TMC, Portugal), and pH_total_ (VWR pHenomenal pH 1100H, connected to a glass electrode calibrated with TRIS-HCl and 2-aminopyridine-HCl buffers). Seawater carbonate system speciation was calculated from the total alkalinity (titration) and pH measurements. Bicarbonate and pCO_2_ values were calculated using CO2SYS software. Seawater parameters are summarized in ESM (Supplementary Table S1).

### Experimental Parasite Infection

We used controlled parasite infections. Gnathiids, namely *Gnathia aureamaculosa,* were obtained from a parasite culture maintained at Lizard Island Research Station since 2001 (Hutson et al., 2018; Grutter et al., 2020). Gnathiids were collected by moving a black tray around the culture tank to simulate the movement of a host, which briefly attracts the unfed (determined by presence of an unengorged gut) gnathiids to the tray and be easily captured with a pipette. After 10 days of acclimation to the CO_2_ treatment, individual damselfish were placed in individual containers (1 L), filled with water from the respective CO_2_ treatment, and randomly assigned to a parasite treatment (parasite, *n* = 48, or control *n* = 45). Six gnathiids were added to the parasite treatment containers. We counted the number of gnathiids attached to each fish at 15 min intervals for a total of 240 min (as at that time, most parasites had detached from the fish, Supplementary Figure S1). Control fish spent the same time in the treatment containers without parasites. The 10-days acclimation ensured that any recent effects of gnathiid parasites were due to this experimental parasite infection and not gnathiids obtained in the wild, as gnathiids only feed on hosts for a few minutes to hours before detaching (Grutter 2003). Following this, the fish were transferred to a hand net, which resulted in any remaining cultured gnathiid parasite being removed from their body as they readily detach when disturbed (Grutter, 1995). Then fish were subjected to an aerobic metabolism analysis described as follows.

### Aerobic Metabolism

Oxygen uptake rates (*Ṁ*O_2_), corrected for fish mass, were determined by using an intermittent flow respirometry, according to the established respirometry methods (Clark et al., 2013; Roche et al., 2013; Rummer et al., 2016). The aerobic metabolism measures were performed between 7:00 and 19:00 h with two runs a day, where four fish were measured simultaneously in each run. The overall aerobic metabolism analysis took 12 days to complete (4 fish per run, two runs a day, eight fish per day). The fish were starved for 24 h prior testing to ensure a postabsorptive metabolic state (Niimi and Beamish, 1974). Maximum metabolic rate (*Ṁ*O_2Max_) was measured by chasing the fish for 3 min in a circular container (30 cm diameter and 10 cm height) with a hand net, followed by air exposure (1 min) involving holding the fish in a hand net. Immediately following air exposure, the fish were gently placed into individual respirometry chambers (volume: 668 ml, including tubing), which were submerged in a water bath (volume: 100 L) containing the respective treatment water. The water bath had a continuous flow of treatment water to ensure that CO_2_ and temperature were kept constant. Water mixing within each chamber was achieved with an external DC brushless pump that moved water through the chamber and around an external circuit of gas-tight tubing (flowrate: 100 ml min^−1^). Automated flush pumps supplied the chambers with oxygenated clean water for 3 min every 8 min, so that the O_2_ levels within the chambers never fell below 80% air saturation. The flush pumps were controlled *via* Profilux 3.1N using a programmed timing sequence. In each chamber, every 2 s, the temperature-compensated oxygen concentration (mg L^−1^) of the water was recorded using the flow-through cells with an integrated optical oxygen sensor (OXFTC2, PyroScience, Germany), calibrated before each run. The flow-through cells were linked to the external circuit of gas-tight tubing and connected to a Firesting Optical Oxygen Meter (PyroScience, Germany) with fiber-optic cables. *Ṁ*O_2Max_ was measured before the first flush, for 5 min after the fish was placed in the chambers. The fish remained in chambers to recover to their resting oxygen uptake rates (resting metabolic rate, *Ṁ*O_2Rest_) over 6 h. *Ṁ*O_2Rest_ is here defined as the average rate of metabolism, when an animal is in a postabsorptive state as mostly inactive (Killen et al., 2014; Metcalfe et al., 2016). *Ṁ*O_2Rest_ was calculated using the average of the three lowest *Ṁ*O_2_ values obtained within 6 h (these values were normally obtained towards the end of the reading, and at least after 3 h of reading). This methodology was previously demonstrated valid for this species as these fish can recover from exercise (after chasing) within 90 min, with their oxygen uptake remaining stable for 8 h (Killen et al., 2014). Moreover, while confined in a respirometer, following recovery, the damselfish remained mostly inactive (Killen et al., 2014). Before and after each fish *Ṁ*O_2_ measurement, the full setup was cleaned with fresh water and the chambers walls wiped with a tissue soaked in 70% ethanol, followed by refilling with seawater; a 1 h of full respirometry cycles was then run with empty chambers to measure any background *Ṁ*O_2_ by bacteria and other microorganisms. Background respiration was assumed to increase linearly (from start to end of each respirometry trial) and was subtracted from each measure of *Ṁ*O_2_. The respirometry data was analyzed with the R software package “respR” (Harianto et al., 2019). The oxygen data was corrected for fish mass and was analyzed as mg O_2_·g_fish_^−1^·hour^−1^. Individual’s capacity to transport oxygen, relative to their baseline rate of oxygen uptake, was determined using both absolute aerobic scope (AAS) and factorial aerobic scope (FAS), which are calculated by subtracting *Ṁ*O_2Rest_ from *Ṁ*O_2Max_ or by the ratio of *Ṁ*O_2Max_ to *Ṁ*O_2Rest_, respectively. AAS is more widely used (and stable) for when the variability in *Ṁ*O_2Rest_ is the major concern (e.g., when animals are restlessness) and FAS is more widely used (and stable), when *Ṁ*O_2Max_ is the main concern (e.g., when animals are averse to be active in a laboratory setting) (Halsey et al., 2018). According to the best practices suggested by Halsey et al. (2018), both AAS and FAS are presented and analyzed. Both AAS and FAS used the mass corrected data.

### Statistical Analysis

All measurements were taken from distinct samples (i.e., each fish). We evaluated differences in the mean number of gnathiids attached during the experimental parasite infection, using a GLMM with Gaussian error distribution, CO_2_, and cleaner present as categorical fixed factors and tank and reef ID as random factors (ESM Supplementary Table S2). We analyzed the differences in size (standard length, SL) and weight using the generalized linear mixed-effect models (GLMM) with Gaussian distribution, CO_2_ treatment, cleaner presence, and parasite treatment as categorical fixed factors, and tank and reef ID as random. For the metabolic data (*Ṁ*O_2Max_, *Ṁ*O_2Rest_, AAS and FAS), we used a gamma error distribution as the metabolic data is right skewed, following Bolker (2008). These models used CO_2_ treatment, cleaner presence, and parasite treatment as categorical fixed factors, and tank replicate, reef ID and measurement time of day (morning vs. afternoon) as random factors (ESM Supplementary Tables S3,S4). The full models, with all possible interactions, were tested using the function “glmmTMB” from the package “glmmTMB” (Brooks et al., 2017) and the function “Anova” from the package “car” (Fox and Weisberg, 2011) in R, version 3.4.3 (R Core Team, 2017). Post-hoc multiple comparisons were performed using the package “emmeans” and applying Tukey corrections *via* the Monte Carlo approximation of the multivariate distribution (Lenth, 2020). Model assumptions and performance were validated using the package “performance” (Lüdecke et al., 2021). Data exploration used the HighstatLibV10 R library from Highland Statistics (Zuur et al., 2009; Zuur et al., 2010).

## Results

The size (SL) of damselfish did not differ with treatment (parasite: χ^2^ = 0.855, d.f. = 1, *p* = 0.355; cleaner: χ^2^ = 0.015, d.f. = 1, *p* = 0.902; and CO_2_: χ^2^ = 0.026, d.f. = 1, *p* = 0.873) nor any interactions of treatments (parasite × CO_2,_ × cleaner: χ^2^ = 0.034, d.f. = 1, *p* = 0.853; parasite × CO_2_: χ^2^ = 0.059, d.f. = 1, *p* = 0.808; parasite × cleaner: χ^2^ = 0.67, d.f. = 1, *p* = 0.413; and CO_2_ × cleaner: χ^2^ = 0.512, d.f. = 1, *p* = 0.474). Likewise, fish weight did not differ with treatment (parasite: χ^2^ = 0.832, d.f. = 1, *p* = 0.362; cleaner: χ^2^ = 0.031, d.f. = 1, *p* = 0.861; CO_2_: and χ^2^ = 0.603, d.f. = 1, *p* = 0.438) nor any interaction of treatments (parasite × CO_2,_ × cleaner: χ^2^ = 1.767, d.f. = 1, *p* = 0.184; parasite × CO_2_: χ^2^ = 0.1029, d.f. = 1, *p* = 0.31; parasite × cleaner: χ^2^ = 1.241, d.f. = 1, *p* = 0.265; and CO_2_ × cleaner: χ^2^ = 0.569, d.f. = 1, *p* = 0.451).

**FIGURE 2:**
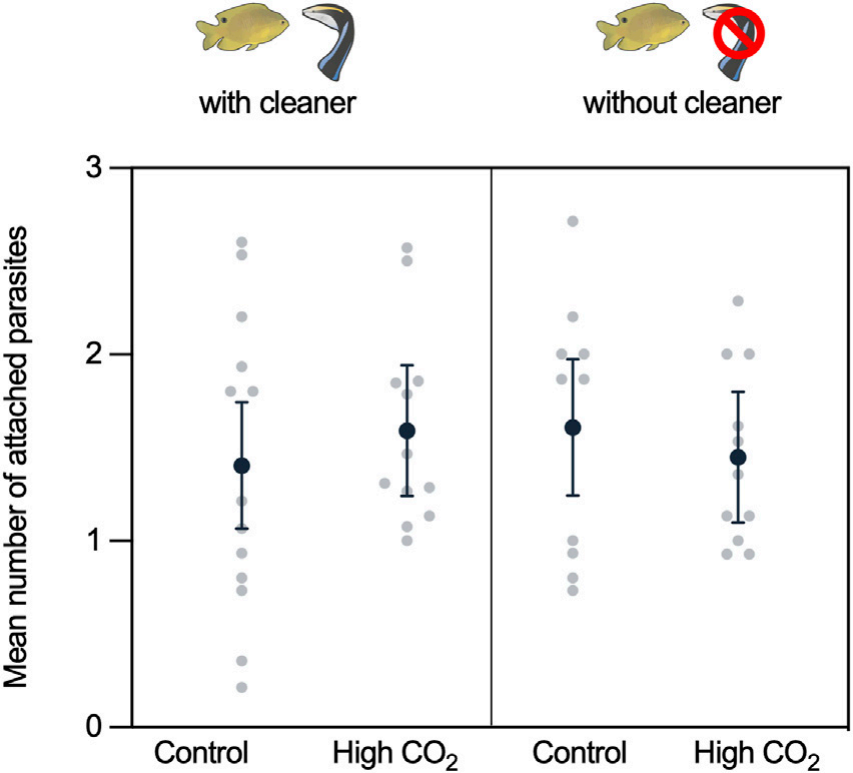
Mean number of attached gnathiids during the experimental parasite infection. Back-transformed predicted means ±95% confidence intervals (C.I.) from the model and raw data values are presented.

During the experimental parasite infection, the damselfish were infected with an average of 1.37 ± 0.08 gnathiids (mean ± SE). This number is within the range they are infected within the wild over 12 h (Grutter et al., 2018). The average number of attached gnathiids did not differ with CO_2_ or cleaner treatment or the interaction between CO_2_ and cleaner treatment (CO_2_: χ^2^ = 0.067, d.f. = 1, *p* = 0.796; cleaner: χ^2^ = 0.031, d.f. = 1, *p* = 0.861; and cleaner × CO_2_: χ^2^ = 0.971, d.f. = 1, *p* = 0.324; Supplementary Table S2, Figure 2).

**FIGURE 3:**
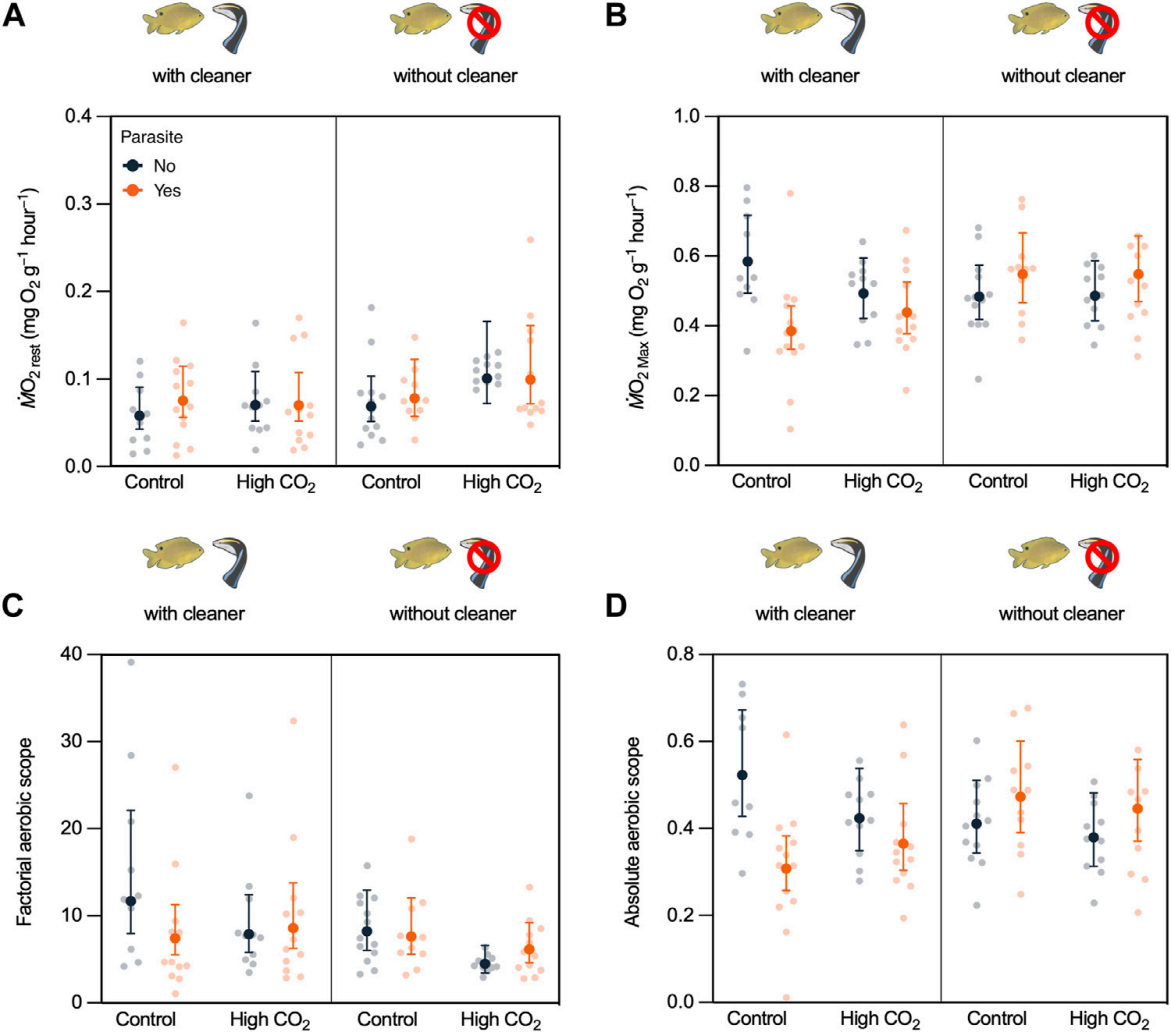
Damselfish *Pomacentrus amboinensis* aerobic physiology obtained through the exhaustive chase protocol according to CO_2_ treatment (Control vs. High CO_2_), cleaner wrasse removal treatment (with cleaner *vs*. without cleaner), and parasite infection (Yes vs. No). **(A)** resting metabolic rate (*Ṁ*O_2Res_, **(B)** maximum metabolic rate (*Ṁ*O_2Max_), **(C)** factorial aerobic scope (FAS), and **(D)** absolute aerobic scope (AAS). Back-transformed predicted means ±95% confidence intervals (C.I.) from the model and raw data values are presented.

Resting metabolism (*Ṁ*O_2Rest_) of fish was not affected by CO_2_, cleaner, or parasite treatment (CO_2_: χ^2^ = 2.289, d.f. = 1, *p* = 0.130; cleaner: χ^2^ = 2.427, d.f. = 1, *p* = 0.119; and parasite: χ^2^ = 0.484, d.f. = 1, *p* = 0.487; Supplementary Table S3, Figure 3A). All interactions were not significant (Supplementary Table S3).

For the maximum metabolic rate (*Ṁ*O_2Max_), there was a significant interaction between the cleaner presence and parasite infection (cleaner × parasite: χ^2^ = 11.390, d.f. = 1, *p* < 0.001, Supplementary Table S3, Figure 3B). *Ṁ*O_2Max_ decreased when damselfish from the cleaner-present reefs were infected with parasites (t-ratio = -3.197; *p* = 0.011; Supplementary Table S4, see Table 1), but not on damselfish from the cleaner-absent reefs (t-ratio = 1.518; *p* = 0.432; Supplementary Table S4, see Table 1). Moreover, *Ṁ*O_2Max_ was lower for damselfish infected with parasites from the cleaner-present reefs compared to infected damselfish from the cleaner-absent reefs (t-ratio = 3.369; *p* = 0.006; Supplementary Table S4, see Table 1). CO_2_ did not affect *Ṁ*O_2Max_ (CO_2_: χ^2^ = 0.026, d.f. = 1, *p* = 0.872, Supplementary Table S3, Figure 3B, see Table 1).

**TABLE 1:** Summary of results of the post-hoc multiple comparisons in relation to the effect of cleaner fish treatment (presence or absence).

Response measured	Significant interaction	Interaction *p*-value	Significant paired test	Cleaner presence effect on response measured	Paired test *p*-value
*Ṁ*O_2Max_	Cleaner × Parasite	<0.001	Cleaner-present, No-parasite *vs.*	-	0.011
			Cleaner-present, Yes-parasite		
*Ṁ*O_2Max_	Cleaner × Parasite	<0.001	Cleaner-present, Yes-parasite *vs.*	-	0.006
			Cleaner-absent, Yes-parasite		
FAS	CO_2_ × Parasite	0.008	Control CO_2,_ No-Parasite *vs.*	NA	0.035
			High CO_2,_ No-parasite		
AAS	Cleaner × Parasite	<0.001	Cleaner-present, No-parasite *vs.*	-	0.011
			Cleaner-present, Yes-parasite		
AAS	Cleaner × Parasite	<0.001	Cleaner-present, Yes-parasite *vs.*	+	0.015
			Cleaner-absent, Yes-parasite		

“+” and “−” represent an increase and decrease, respectively, in the response measured.

Regarding the factorial aerobic scope (FAS), there was a significant interaction between CO_2_ and parasite infection (CO_2_ × parasite: χ^2^ = 6.9591, d.f. = 1, *p* = 0.008, Supplementary Table S3, Figure 3C). Post-hoc comparisons indicated that nonparasite damselfish under high CO_2_ had significantly lower FAS than that of nonparasite fish in control CO_2_ (t-ratio = -2.759; *p* = 0.035; Supplementary Table S4, see Table 1). Cleaner presence did not affect FAS (FAS: χ^2^ = 3.528, d.f. = 1, *p* = 0.060, Supplementary Table S3, Figure 3C, see Table 1).

Absolute aerobic scope was altered by an interaction between cleaner presence and parasite infection (cleaner × parasite: χ^2^ = 11.414, d.f. = 1, *p* < 0.001, Supplementary Tables S4, S5, Figure 3D), but high CO_2_ had no significant effect (CO_2_: χ^2^ = 0.580, d.f. = 1, *p* = 0.476, Supplementary Table S3, Figure 3D). Post-hoc comparisons revealed that parasite infection decreased AAS only in fish with access to cleaners (t-ratio = -3.191; *p* = 0.011; Supplementary Table S4, see Table 1) and that parasite damselfish on cleaner-absent reefs had higher AAS than parasitized damselfish from the cleaner-present reefs (t-ratio = 3.063; *p* = 0.015; Supplementary Table S4, see Table 1).

## Discussion

Cleaner wrasses provide an essential ecological service to their clients by controlling their ectoparasite loads through direct removal of ectoparasites from their surfaces and reducing infection rates by lowering gnathiid densities (Grutter, 1996; Grutter, 1999; Grutter et al., 2018). We hypothesized that clients deprived of access to cleaning services would be physiologically adapted to deal with ectoparasite infections. In line with this hypothesis, we found that the ectoparasite infection results in a decreased maximum metabolic rate [*Ṁ*O_2Max_, inducing so-called limiting stress, as defined in Heuer and Grosell, 2014)] with parasitism, only in ambon damselfish from the cleaner-present reefs. As infected damselfish of cleaner-present reefs had lower *Ṁ*O_2Max_ than infected damselfish from cleaner-absent reefs (Figure 3B), this suggests that damselfish from the cleaner-absent reefs are more adapted to tolerate ectoparasite infection. Parasite infection might be lowering *Ṁ*O_2Max_ due to the elevated costs of activation of an immune response. In mice, activation of the immune response led to a decrease in *Ṁ*O_2Max_, and the selection for animals with higher *Ṁ*O_2Max_ led to a reduction in the innate immune function (Downs et al., 2013). Even low parasite infections can have huge costs, especially for the small hosts like damselfish at settlement, as gnathiids can kill their hosts, or by consuming 85% of their blood, which can cause significant sublethal effects (Clague et al., 2011; Waldie et al., 2011). Therefore, in reefs where cleaners are absent, fish might either develop a higher tolerance to gnathiid infection (e.g. better immune response) or be exposed to strong selective pressure for a tolerance to gnathiids. Indeed, at settlement stage, gnathiid infection can increase damselfish oxygen consumption (resting metabolic rate) and decrease their critical swimming speed, making damselfish more prone to predation (Grutter et al., 2011). Previously, Grutter et al. (2011), suggested that if gnathiids affect the settlement stage damselfishes, they might ultimately affect the adult population distribution and abundance. The observed tolerance to ectoparasite infection observed in damselfishes from the cleaner-absent reef might indeed reflect this, where, due to the absence of cleaner fishes and high parasitism, only individuals that tolerate parasite infections could successfully settle and reach adulthood. Variation of tolerance to ectoparasitism in wild fish populations has been previously described in dace (*Leuciscus leuciscus*) (Blanchet et al., 2010). This variation could be due to an investment in limiting tissue degradation during ectoparasite feeding, such as investing in higher cell regeneration (i.e. better immune response, Råberg et al., 2009), or it could be a selection for individuals that are genetically better at dealing with infection (Klemme et al., 2020). However, the mechanisms that could provide higher parasite tolerance to damselfishes from the cleaner-absent reefs observed here remain unknown and need to be further investigated. In contrast, in reefs without cleaners, selection might favor damselfish that suppress their innate immune function, preventing a limitation on their aerobic capacity (*Ṁ*O_2Max_), as described in mice selected for higher *Ṁ*O_2Max_ (Downs et al., 2013).

*Ṁ*O_2Rest_ was not altered by parasite infection regardless of its access to cleaning or CO_2_. Infection with small ectoparasites in salmon also had no effects on *Ṁ*O_2Rest_ (Hvas et al., 2017). On the other hand, studies using larger parasites, such as *Anilocra* spp*.* isopods*,* showed enhanced resting metabolic rate in the coral reef fish (Östlund-Nilsson et al., 2005; Binning et al., 2013). Yet, it is worth mentioning that such an increase in the resting metabolic rate was due to the destabilizing effect of the asymmetrically attached parasite rather than any physiological effect of parasitism. This indicates that parasite infection does not add a so-called loading stress (as defined in Heuer and Grosell 2014). Moreover, as found by Couturier et al., 2013, exposure to high CO_2_ did not affect the damselfish resting metabolic rate (*Ṁ*O_2Rest_), suggesting that the metabolic costs of living in a high CO_2_ environment, namely potentially altered acid-base balance, ion regulation, and cardiorespiratory function also do not add a loading stress for this species (Figure 3A).

When we analyzed damselfish oxygen transport capacity relative to their resting rate of oxygen uptake (i.e., aerobic scope), we observed that their response to parasite infection was dependent on access to cleaning services. Only fish from the cleaner-present reefs decreased AAS (*Ṁ*O_2Max_ - *Ṁ*O_2Rest_) with parasite exposure. This suggests that fish who lack access to cleaning services have a higher physiological tolerance to parasite infection. Yet, independent of cleaner presence and only in fish without parasite infection, FAS (*Ṁ*O_2Max_/*Ṁ*O_2Rest_) decreased with CO_2_ (but not AAS). Here, fish under control conditions without parasite infection had the highest average FAS and CO_2_ significantly decreased damselfish FAS. This indicates that the combination of parasite stress and CO_2_ does not have an additive effect. However, by comparing FAS with both *Ṁ*O_2Max_ and *Ṁ*O_2Rest_, we observed that this decrease in FAS results from different physiological processes for both the stressors. On one hand, the decrease in FAS under high CO_2_ could be driven by a slight (nonsignificant) increase in *Ṁ*O_2Rest_, suggesting that a slight increase in resting metabolic costs (i.e., increase in loading stress) under high CO_2_ is what is causing a lower damselfish aerobic capacity (FAS). On the other hand, the decrease in FAS (and AAS) due to parasite infection is driven by a decrease in *Ṁ*O_2Max_, possibly due to an increase in the damselfish immune response. The difference between FAS and AAS is related to their different sensitivities for each metabolic parameter measured. While both FAS and AAS are used to represent an animal’s capacity to increase its aerobic metabolic rate above the maintenance levels, AAS is more widely used for when the variability in *Ṁ*O_2Rest_ is the major concern, and FAS is more widely used when *Ṁ*O_2Max_ is the main concern (Halsey et al., 2018). Following the best practices used in the field, we present both measures. Yet, we must interpret this result with caution as the present exposure to high CO_2_ had no evolutionary component and was of a relatively short duration (Sunday et al., 2014). Therefore, we cannot exclude the possibility that these fishes could be able to adapt to this increase in CO_2_.

Experiments on species’ resilience to climate change stressors should be analyzed in an evolutionary perspective. This study starts with the premise that cleaning interactions could be altered with OA, since Paula and collaborators found significant changes in cleaning motivation after a 45 day acclimation period to high CO_2_ (Paula et al., 2019a). Additionally, in a follow-up study, it was observed that: i) cleaner wrasse populations have a standing variation to deal with mild increases of CO_2_ (i.e., 750 µatm pCO_2_) from a behavioral perspective and ii) only at higher acidification levels (980 µatm pCO_2_) was their cognitive performance severely affected (Paula et al., 2019b). However, one should note that to fully address the climate change problem, we should consider a multistressor perspective, e.g., including a temperature increase (Gunderson et al., 2016). Within this context, it is worth noting that both the cleaner and clients are particularly sensitive to extreme heatwave events (e.g., 2016 marine heatwave and associated coral bleaching), as their abundance can diminish drastically after such extreme events (Triki et al., 2018; Triki and Bshary, 2019). Gnathiid abundance also decreased at our study site during the same extreme event (2016), and to a following one in 2017. But while it quickly recovered after the first extreme event (possibly due to lower coral cover, see Paula et al., 2021), it did not do so in 2017 and remained low postbleaching (in 2018) (Sikkel et al., 2019). This overall decrease in gnathiids may have been caused by an interaction between the short-term negative impacts of thermal stress on gnathiids, as shown in laboratory studies (Shodipo et al., 2020), and a decline in host availability, causing gnathiid abundance to drop (Sikkel et al., 2019; Triki and Bshary, 2019). Since heatwave intensity and frequency is increasing (Oliver et al., 2018), client fish (e.g., *P. amboinensis*) population attempts of adaptation to either ocean acidification or parasite infection can quickly be erased following such extreme events, if, for example, individuals that develop tolerance to either parasite infection or OA die during such extreme events. Additionally, this possible adaptation to parasite infection (e.g., putative higher investment in higher cell regeneration) may come at a cost, as damselfish from the cleaner-absent reefs are smaller, and thus probably less fecund, since fecundity and size are correlated in these fish (Birkeland and Dayton 2005; Clague et al., 2011).

In conclusion, we demonstrated, for the first time, that cooperative cleaning interactions, fundamental ecological components of coral reef fish ecosystems, can influence the physiological fitness of a client fish species. Namely, we found that the lack of access to cleaning services leads to more physiological tolerance to parasite infection. However, independent of access to cleaners, high CO_2_ lowered fish fitness, although this was not exacerbated by parasite infection. This study adds a layer of complexity to the climate change-related studies, namely the importance of species interactions, that should be included to fully understand the biological impacts of climate change in the ocean of tomorrow.

## Data Availability

The datasets generated and analysed during this study are available in the Figshare repository, https://doi.org/10.6084/m9.figshare.13656647.
