# Supplementary material for: Access to Cleaning Services Alters Fish Physiology Under Parasite Infection and Ocean Acidification
Source: Front Physiol. 2022 Jun 8;13:859556. doi: 10.3389/fphys.2022.859556 (PMC9213755; doi:10.3389/fphys.2022.859556)
Supplement: Supplementary file 1 [file DataSheet1.pdf]

Supplemental materials

Figure S1. Number of adult cleaner fish observed in the long-term cleaner fish removal experiment at Lizard Island. Dots are individual reef values and lines are means.

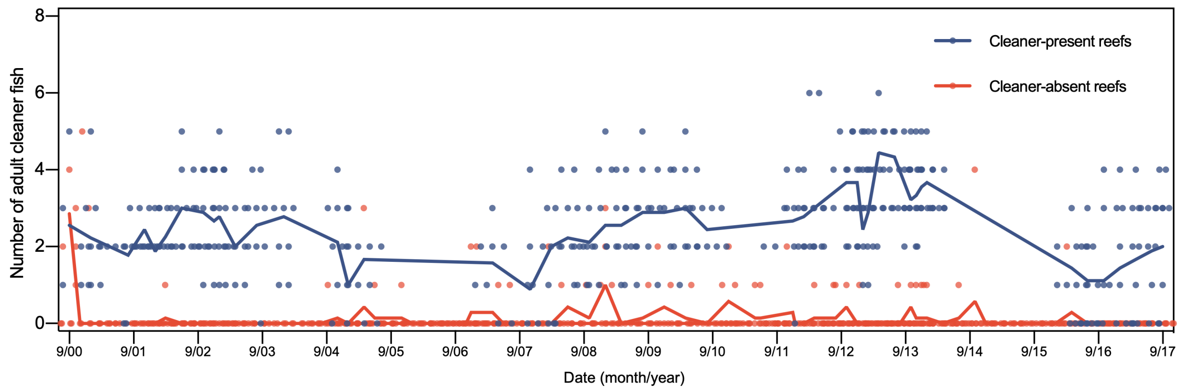

Figure S2. Number of parasites attached to damselfish (mean ± std. error) according to cleaner presence and CO_2 treatment during the parasite infection assay.

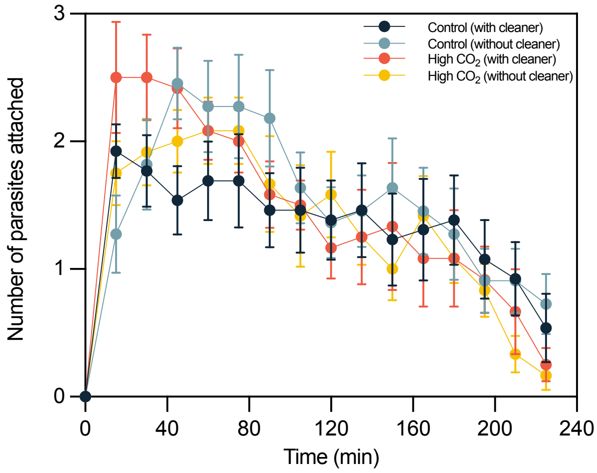

Table S1. Seawater physicochemical parameters in all experimental setups (mean ± SE). Salinity, pH, temperature and alkalinity were measured daily over the 23 days of the experiment and averaged over the whole experimental period. The combination of total alkalinity (TA) and pH was used to calculate carbonate system.

System	*A1*	*A2*	*A3*	*A4*	*C1*	*C2*	*C3*	*C4*
**Measured**								
Temp. (°C)	24.8 ± 0.8	24.8 ± 0.8	24.8 ± 0.7	24.9 ± 0.7	24.7 ± 0.7	24.7 ± 0.8	24.7 ± 0.7	24.7 ± 0.8
pH	7.69 ± 0.04	7.67 ± 0.04	7.67 ± 0.04	7.67 ± 0.06	8.05 ± 0.02	8.06 ± 0.02	8.06 ± 0.02	8.06 ± 0.02
TA (µmol/kgSW)	2115.7 ± 15.3	2115.6 ± 15.1	2116.5 ± 15.2	2115.9 ± 16.5	2110.3 ± 12.9	2110.3 ± 12.9	2110.3 ± 12.9	2110.3 ± 12.9
Salinity (ppt)	35	35	35	35	35	35	35	35
**Calculated**								
TC (µmol/kgSW)	1992.5 ± 23.2	1998.1 ± 18.9	2003.1 ± 23.5	2000 ± 26.6	1821.7 ± 17.1	1819.3 ± 17.2	1817.8 ± 16.4	1815.3 ± 18.4
pCO2 (µatm)	938.1 ± 101	969 ± 84.5	1000.7 ± 106.2	990.8 ± 125.9	348.2 ± 20.1	343.8 ± 19	341.9 ± 19.7	338.3 ± 21.6
Ω Arg	1.59 ± 0.16	1.54 ± 0.13	1.51 ± 0.16	1.53 ± 0.21	3.15 ± 0.15	3.17 ± 0.15	3.19 ± 0.15	3.21 ± 0.17

Table S2. Analysis of deviance table (Type II tests) of mean number of parasites for CO_2 and cleaner presence treatment for generalized linear mixed-effects model (GLMM). Values mentioned in main text are italicized.

	Mean number of parasites		
	χ2	*D.f.*	*p*
Cleaner	0.031	1	*0.861*
CO_2	0.067	1	*0.796*
Cleaner × CO_2	0.971	1	*0.324*

Table S3. Analysis of deviance table (Type II tests) of metabolic physiology measures [resting metabolic rate ($\dot{M}O_{2Rest}$), maximum metabolic rate ($\dot{M}O_{2Max}$), factorial aerobic scope (FAS) and absolute aerobic scope (AAS)] among CO_2 treatments, cleaner presence, and parasite infection for generalized linear mixed-effects models (GLMM). Values of p<0.05 are shown in bold. Values mentioned in main text are italicized.

	$\dot{M}O_{2Rest}$			$\dot{M}O_{2Max}$			FAS			AAS		
Predictors	χ^2	*D.f.*	*p*	χ^2	*D.f.*	*p*	χ^2	*D.f.*	*p*	χ^2	*D.f.*	*p*
Cleaner	2.427	1	0.119	1.496	1	0.221	3.528	1	0.060	0.580	1	0.446
CO_2	2.289	1	0.130	0.026	1	0.872	2.656	1	0.103	0.507	1	0.476
Parasite	0.484	1	0.487	0.990	1	0.320	0.879	1	0.348	1.102	1	0.294
Cleaner × CO_2	0.775	1	0.379	0.009	1	0.926	1.651	1	0.199	0.201	1	0-654
Cleaner × Parasite	0.108	1	0.743	11.390	1	***<0.001***	2.976	1	0.085	11.414	1	***<0.001***
CO_2 × Parasite	0.924	1	0.337	1.332	1	0.248	6.959	1	***0.008***	1.718	1	0.190
Cleaner × CO_2 × Parasite	0.141	1	0.707	1.909	1	0.167	0.034	1	0.853	1.508	1	0.219

Table S4. Summary output for the post-hoc pairwise comparisons of metabolic physiology measures [maximum metabolic rate ($\dot{M}O_{2Max}$), factorial aerobic scope (FAS) and absolute aerobic scope (AAS)] among CO_2 treatments, cleaner presence and parasite infection. P-values were adjusted using tukey corrections. Values of p<0.05 are shown in bold. Yes = yes parasites, No = no parasites.

**$\dot{M}O_{2Max}$**				
Post-hoc comparison	*estimate*	*Std. error*	*t-ratio*	*p-value*
with cleaner, No - without cleaner, No	-0.194	0.162	-1.198	0.630
with cleaner, No - with cleaner, Yes	-0.567	0.177	-3.197	***0.011***
with cleaner, No - without cleaner, Yes	0.045	0.158	0.287	0.992
without cleaner No - with cleaner, Yes	-0.373	0.182	-2.048	0.179
without cleaner No - without cleaner, Yes	0.240	0.158	1.518	0.432
with cleaner, Yes- without cleaner, Yes	0.613	0.182	3.369	***0.006***
**FAS**				
Post-hoc comparison	*estimate*	*Std. error*	*t-ratio*	*p-value*
Control, No - High CO_2, No	-0.071	0.026	-2.759	***0.035***
Control, No - Control, Yes	-0.029	0.015	-1.929	0.224
Control, No - High CO_2, Yes	-0.036	0.023	-1.573	0.399
High CO_2, No - Control, Yes	0.042	0.027	1.557	0.409
High CO_2, No - High CO_2, Yes	0.035	0.022	1.630	0.368
Control, Yes- High CO_2, Yes	-0.007	0.024	-0.272	0.993
**AAS**				
Post-hoc comparison	*estimate*	*Std. error*	*t-ratio*	*p-value*
with cleaner, No - without cleaner, No	-0.397	0.247	-1.603	0.383
with cleaner, No - with cleaner, Yes	-0.857	0.269	-3.191	***0.011***
with cleaner, No - without cleaner, Yes	-0.043	0.230	-0.185	0.998
without cleaner No - with cleaner, Yes	-0.461	0.281	-1.640	0.362
without cleaner No - without cleaner, Yes	0.354	0.244	1.449	0.473
with cleaner, Yes- without cleaner, Yes	0.815	0.266	3.063	***0.015***
